# Economic impact of traumatic spinal cord injuries in the United States

**DOI:** 10.20517/2347-8659.2019.15

**Published:** 2019-07-20

**Authors:** Christopher H. Merritt, Matthew A. Taylor, Caleb J. Yelton, Swapan K. Ray

**Affiliations:** Department of Pathology, Microbiology, and Immunology, University of South Carolina School of Medicine, Columbia, SC 29209, USA

**Keywords:** Traumatic spinal cord injury, healthcare resource, American spinal injury association grade, hospital length of stay, rehabilitation, post-injury employment

## Abstract

Individuals having sustained traumatic spinal cord injury (TSCI) in the United States are living longer as compared to historical trends, thanks to an ever-evolving understanding of the nature of this injury. Despite this, multiple barriers to care for TSCI patients remain including variations in government-issued veteran insurance, privatized insurance, and among uninsured individuals. The United States alone experiences 12,000 new TSCI cases every year, many of these are found to occur in a growing proportion of elderly individuals. It is crucial to understand both the short-term direct costs as wells as the long-term rehabilitation costs required by these TSCI patients. The lifetime financial burden for those having sustained a TSCI can be immense for patients, insurance companies, and hospital systems alike. Among those with TSCI, re-hospitalization rates are high, leading to increased healthcare resource utilization within this specific patient population. Costs can quickly balloon into hundreds of thousands of dollars and cause a profound financial burden for these patients. This review article seeks to communicate an understanding of the current financial landscape surrounding TSCI patients. The authors will also examine the costs of acute emergency room surgical care such as American spinal injury association grade, hospital length of stay, as well as the timing delay between injury and surgical decompression. Long-term costs associated with TSCI such as rehabilitation, care of secondary comorbidities, and post-injury employment prospects will be examined as well. These costs will be framed from the patient’s perspective as well as from both the hospital and insurance company’s perspectives. It is hoped a complete understanding as to what makes TSCI such a medically and financially burdensome injury will allow for improved healthcare resource utilization in this population.

## INTRODUCTION

To begin a discussion of the economic impact of traumatic spinal cord injury (TSCI), it becomes necessary to first have an understanding of the epidemiology and disease burden of TSCI. TSCIs are sustained following major traumatic events, such as falls, motor vehicle accidents, or acts of violence. TSCIs are life-changing, economically impactful traumas that are estimated globally to affect 13 new individuals per 100,000 per year. But this incidence was found to double in North America, affecting nearly 26 new individuals per 100,000 per year^[1]^. The National Spinal Cord Injury Statistical Center, which is both the longest running and largest database containing the specifics of TSCIs in the United States, estimates the incidence of new TSCIs to be roughly 40 cases per one million in the United States, or roughly about 12,000 new cases per year^[2]^. The incidence of TSCI in the United States has historically been held constant, with the largest increases in incidence being observed in the elderly population in the specific context of an increase in the number of falls as an individual ages^[3,4]^. The prevalence of TSCI in the United States is estimated to be approximately 273,000, within a range of 238,000 to 332,000^[2]^. Within the prevalent population as a whole, more severe injuries were observed in younger individuals as compared to those living to older age with incomplete and/or lower level injuries with resulting high degrees of independence^[5]^. The average age at the time of spinal cord injury is estimated to be 42.6 years of age with males accounting for 80.7% of new cases, vastly outnumbering their female counterparts.

**Figure 1.**
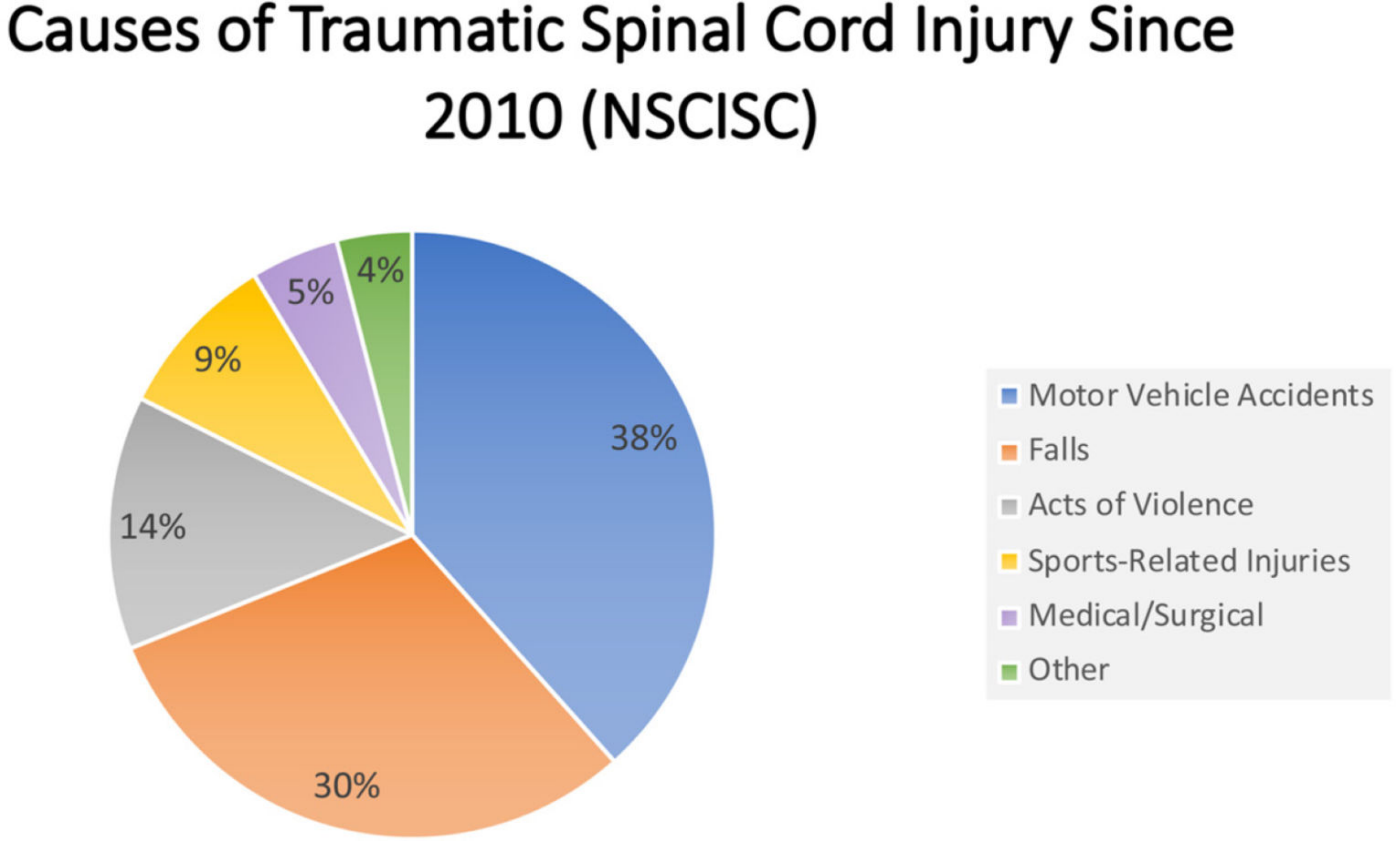
A pie chart illustrating the major causes of TSCI since 2010^[2]^ according to the NSCISC. The NSCISC estimates that the most common causes of TSCI include motor vehicle accidents (blue), mechanical falls (orange), and acts of violence (gray). Less commonly TSCI is caused by sports-related injuries (yellow), medical/surgical causes (pink), and other miscellaneous causes not previously listed (green) TSCI: traumatic spinal cord injury; NSCISC: National Spinal Cord Injury Statistical Center

**Figure 2.**
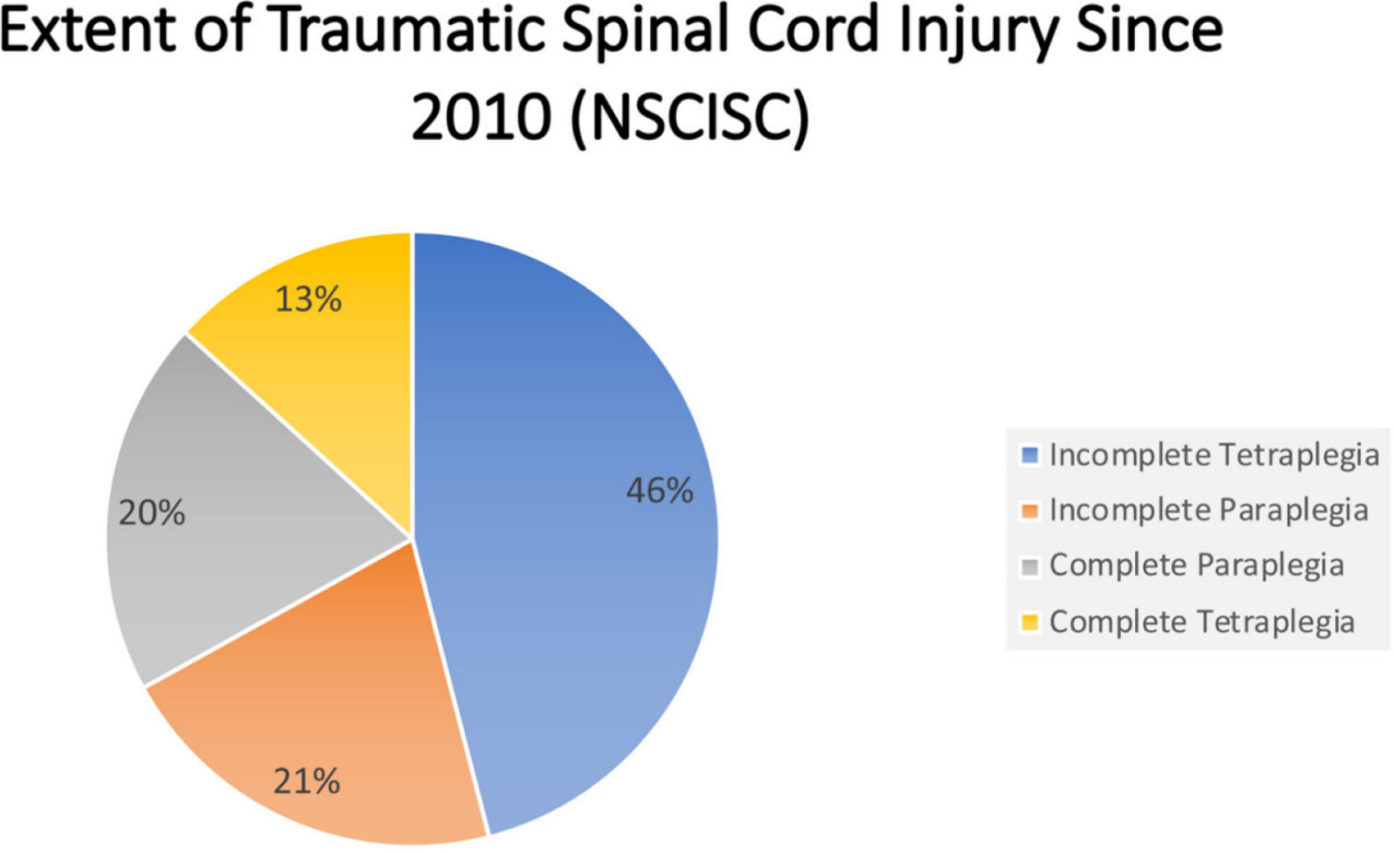
A pie chart illustrating the extent of injury following TSCI since 2010^[2]^ according to the NSCISC. The NSCISC estimated that nearly half of all TSCI resulted in the extent of injury known as incomplete tetraplegia (blue). Incomplete and complete paraplegia were similar in prevalence following TSCI (represented by orange and gray, respectively) while complete tetraplegia (yellow) was the least common extent of injury following TSCI as compared to the other major extent of injury categories. TSCI: traumatic spinal cord injury; NSCISC: National Spinal Cord Injury Statistical Center

Those with TSCIs have recently been found to be living longer, when compared to historical trends^[6]^. Vehicle crashes remain the leading cause of injury, followed by falls, and then acts of violence (i.e., gunshot wounds) [Figure 1]^[2]^. The neurological deficits sustained following a TSCI are categorized by its corresponding American Spinal Injury Association (ASIA) score, ranging from A to E with A indicating profound deficit and E indicating normal function[Table 1]^[7]^. The extent of injuries varies as well, with incomplete tetraplegia being the leading extent of injury, followed by incomplete paraplegia, complete paraplegia, and finally complete tetraplegia[Figure 2]^[2]^. The limitations on an individual’s activities of daily living were found to be largely determined by the location and completeness of the injury sustained, where total hours of care were dependent upon injury level and severity^[8]^. TSCIs exact a heavy financial burden both in the acute care setting as well as within the context of longer-term rehabilitation that often follows the initial injury^[9]^. The costs associated with TSCIs are greatly affected by both the patient’s extent of injury and subsequent degree of disability. Unsurprisingly, the overall life expectancy for those individuals sustaining a TSCI remain significantly below the average life expectancy in the United States^[10]^. An understanding of the epidemiological burden of TSCI in the United States warrants a further discussion on the cost, reimbursement, and subsequent disability associated with such an economically, medically, and psychologically impactful event.

## ACUTE CARE COSTS FOLLOWING TRAUMATIC SPINAL CORD INJURY

Immediately following a TSCI, the vast majority of patients will promptly seek medical care consisting of both surgical stabilization and vertebral decompression^[11–14]^. The high acuity of TSCIs often exacts a heavy financial burden in addition to a life-altering disability for these patients. In the United States, approximately 50% of TSCI patients have their medical costs covered through a private insurer. Medicaid, a state-run medical insurance provider for financially disadvantaged patients, covers 28% of those having sustained a TSCI. The remaining population has their medical costs covered through Medicare or the Veterans Health Administration (VHA)^[13]^. The average cost for the initial injury and recovery phase, termed the *acute phase*, can run $142,366^[12]^. The majority of these charges will be covered through a patient’s primary medical insurance. Most patients, with the exception of eligible military veterans through the VHA, are often left with high co-pays that place an additional undue financial burden on the recovery process.

Evidence demonstrates that surgical intervention within the first 72 h post-injury is both a key prognostic and cost-determining factor in the context of TSCI. Surgical intervention within this crucial window has been shown to directly correlate with a decreased hospital length of stay (LOS) and subsequent decreased medical costs^[15,16]^. If surgical intervention is received within 72 h following the initial injury, hospitals were found to save an average of $14,000 on resource utilization. Additionally, patients were found to have a greater chance of neurological recovery and were spared approximately $45,000 in medical costs. A prospective cohort study investigating the relationship between the delay of surgical decompression following TSCI and neurological recovery found that decompression within the first 24 h more than doubled the chance of recovery of a 2 ASIA grade TSCI as compared to those who received spinal cord decompression outside of this 24 h window^[15,17]^. This rapid surgical turnaround within 24 h was found to be just as safe^[18]^. However, this crucial window presents an access to care issue for those living in rural areas in which there is a high prevalence of TSCI, but low rate of hospitalization with subsequent inflated healthcare costs^[12]^. Sparsely located hospitals in rural areas ill-equipped to manage complex TSCIs may underlie the delayed care observed in rural areas^[12,19]^.

## HOSPITAL LENGTH OF STAY FOLLOWING TRAUMATIC SPINAL CORD INJURY

The average hospital LOS following a TSCI was found to be approximately 12 days, twice as long as patients without TSCI. Interestingly, patients between the ages of 18–29 averaged 13.5 days in the hospital, while elderly patients (over 60 years old) averaged only 10 days. This is a surprising observation that can be attributed to younger age being a major risk factor for more severe forms of TSCI^[12]^. Surgical intervention is often necessary for severe TSCIs and is significantly more expensive than conservative medical management. In a study conducting a cost/benefit analysis in elderly patients with odontoid (C1-C2) fractures, it was found that the cost of surgical intervention was approximately $50,000 per patient, while the cost of medical management alone was more akin to $30,000 per patient. When considering the options between surgical and medical management, it is important to note that patients between the ages of 65–85 had a favorable increase in quality adjusted life years (QALY) following surgical management. These patients’ qualities of life improved following surgical management to offset the high costs of care. Patients over the age of 85 did not see the favorable QALY improvement from surgical intervention, suggesting this population would have the greatest cost-benefit from conservative medical management as compared to surgical intervention^[20]^. The ASIA score can be utilized as a determinant of emergency room (ER) cost as well^[15,21]^. Using this information, TSCI surgical hospital costs can be lowered by trying to target certain age groups (under 85) and by attempting surgical intervention sooner^[22]^.

## RECOVERY AND LONG-TERM DISABILITY FOLLOWING TRAUMATIC SPINAL CORD INJURY

### Post-injury rehabilitation

**Figure 3.**
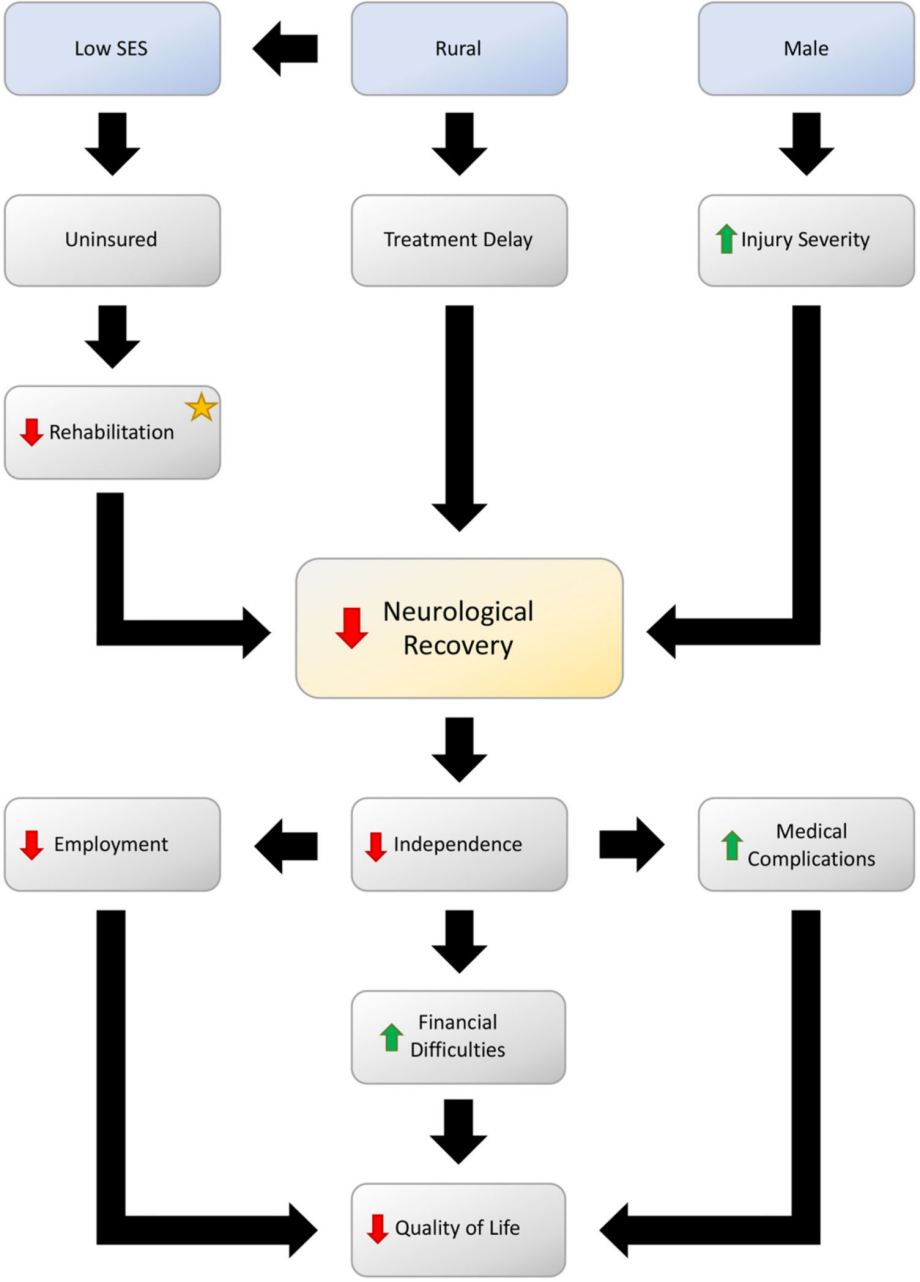
A flowchart illustrates the primary risk factors for a TSCI (blue) and the obstacles TSCI patients may face throughout their lives. Decreased neurological recovery (yellow) is the factor that has the greatest negative impact on a patient. Access to rehabilitation (star) is the only modifiable attribute shown that can reduce the cascade of negative events leading to a decreased patient quality of life. SES: socioeconomic status; TSCI: traumatic spinal cord injury

TSCI recovery is divided into three major phases: acute, post-acute, and chronic^[23,24]^. The acute phase is marked by post-injury care received in the hospital, while post-acute and chronic phases are distinguished through post-injury care delivered in an outpatient setting^[24]^. While the timeframe of each of these phases varies, neurological recovery has been found to occur during the acute and post-acute phases. This crucial recovery period has been found to last between 12–18 months, with the majority of improvement observed in the first 3 months post-injury^[25]^. During the acute and post-acute phases, rehabilitation seeks maximize neurological recovery as measured by the ASIA grade^[26,27]^. A patient will enter the chronic phase when they have reached their maximum neurological recovery; therefore, priorities in the chronic phase shift to minimizing common long-term TSCI co-morbidities and normalizing a patient’s new post-injury standard of living^[28]^. The neurological recovery and the quality of life of the TSCI patients are dependent on various primary risk factors and the obstacles that they may face throughout their lives [Figure 3].

To date, few studies have examined the recovery rates corresponding with the time between TSCI and initiation of rehabilitation^[23]^. Regardless, studies have shown TSCI patients having access to rehabilitation corresponds to better outcomes and a greater chance for patients to reclaim their roles as active members of the society^[29]^. Despite its importance, discrepancies of who should receive rehabilitation continue to exist. A study investigating rehabilitation rates in patients with TSCI examined patients with private insurance, government insurance (Medicare/Medicaid), and the uninsured. Patients with private insurance were referred to rehabilitation services 84.6% of the time, while government and the uninsured were referred rehabilitation 55.5% and 55.2% of the time, respectively, despite both populations having similar injury severities. This study also found that patients with government insurance had an average LOS of 12 days longer than both privatized insurance and those who remain uninsured. However, the explanations are varied. Claridge *et al*.^[28]^ hypothesizes that uninsured patients are simply rejected from most rehabilitation facilities and are inevitably sent home, while privately insured patients are transferred to rehabilitation facilities as soon as possible. Patients with government insurance are kept in the hospital while case management explores potential options, explaining their increased LOS^[28]^. Although not surprising, these results give rise for concern. Increased time between injury and rehabilitation has been associated with decreased long-term quality of life and a decreased ability to live independently; thus, raising the long-term cost of care for these individuals. Rehabilitation teaches patients to prevent secondary health complications, maximizing function and work towards long term healthy lifestyles^[23]^.

### Long-term complications of a traumatic spinal cord injury

In the years following a TSCI, patients face a risk of several severe co-morbidities. Most fatal complications are due to urinary tract infections (UTIs), sepsis due to pneumonia, and pressure ulcers (in those with T1-S5 injuries)^[30,31]^. A medium-sized cohort study found that 47.6% of TSCI participants were treated for a UTI, 33.8% were treated for pneumonias, 27.5% for depression, and 19.7% for a decubitus ulcers^[32]^. Characterized as a “never event”, almost one third of all pressure ulcers are seen in paralyzed patients. The estimated cost for treating a stage IV pressure ulcer (an ulcer that extends into the underlying bone and muscle^[33]^) is approximately $124,000-$129,000 per instance^[34,35]^. Sepsis, the second most expensive of the above listed comorbidities in TSCI patients, was found to cost around $27,000 per stay in the intensive care unit (ICU). When broken down to the cost by day, the cost of sepsis in the ICU per day in the United States was just over $4,500^[36]^. As suggested in the data from the medium sized cohort, TSCI paralysis is a risk factor for increased UTI rates^[32,37]^. The most common of the comorbidities and the least expensive, it cost around $8,300 per hospital treatment^[37]^.

### Post-injury re-hospitalization rates

Patients within the first year following a TSCI are at a significant risk for re-hospitalization. One study estimates a re-hospitalization rate between 36%-45% in the first year post-injury, decreasing to a 30% re-hospitalization risk in subsequent post-injury years^[38]^. The authors of a 2015 study investigating emergency room visits (ERV) and emergency re-hospitalizations (ERH) in chronic TSCI patients found that 37% of participants had at least 1 ERV in the last year, with half of those visits progressing to an ERH^[39]^. The average hospital LOS for these patients was found to be 21 days^[40]^. An additional study found that the only modifiable risk factor for a TSCI patient ERH is lower functional independence following initial rehabilitation^[41]^. Lack of independence is an important issue for uninsured TSCI patients, who encompass 12% of the TSCI population^[11]^. As stated previously, most uninsured TSCI patients forego rehabilitation, causing decreased functional independence and a subsequent increased risk of medical emergencies^[42]^.

TSCI patients re-admitted to a hospital post-injury experience a wide range of costs that are dependent on their co-morbidities. A 2018 study followed a cohort of TSCI patients over a decade while analyzing their use of health care services over that period. This study found that a combined $49.4 million was spent on health care services over this 10-year span for all 303 participants. Interestingly, two-thirds of those costs were utilized by only 16.5% of the study population (termed High Utilizers), with each individual charging $51,860 per year. High Utilizers had an ERH 2.6 times per year with an average LOS of 9.6 days, often being treated for multiple co-morbidities. High Utilizers were commonly male, of a racial minority, of low socio-economic status, with high-grade TSCI, and experienced frequent pressure ulcers. In contrast, 53% of chronic TSCI patients were considered Low Utilizers. These patients on average visited the ED 0.1 times per year and only stayed in the hospital 0.3 days per year^[38]^.

## MILITARY VETERANS SUSTAINING TRAUMATIC SPINAL CORD INJURY

According to the department of veterans affairs, the VHA is the largest network of TSCI care in the country, with over 1,200 integrated healthcare facilities distributed throughout the country. As of 2018, there are over 19 million United States military veterans, and approximately 9.15 million of those veterans are enrolled in the veterans affairs (VA) health care system; making the VHA a healthcare provider for approximately 2.8% of the American population^[43,44]^. In order for a military veteran to qualify for VA-sponsored healthcare, they must have served under active duty and have been honorably discharged. Veterans sustaining a TSCI while in active military service are eligible for monthly disability compensation in addition to the healthcare coverage that all VHA-eligible veterans receive^[45]^. Veterans who are injured in connection to their military service are entitled to comprehensive healthcare coverage with zero monetary responsibility falling onto the patient^[46]^. The VHA provides an interesting perspective on health care resource allocation due to eligible veterans being the sole TSCI population in the United States with no financial responsibility for their post-injury TSCI care.

### Traumatic spinal cord injury costs in the veterans affairs health care system

According to the VHA, there are approximately 26,000 TSCI patients who are eligible to receive VHA-sponsored treatment, half of which chose to undergo specialty treatment within the VA health care system^[45]^. The first 12 months post-injury were found to be the costliest, with the average patient being charged $606,349 within the first year. Patients were then charged an average of $92,454 annually for long-term care^[47]^. However, these charges can vary greatly depending on the severity and extent of the injury. Veterans with C1-C4 tetraplegia accrue an average of $1,064,716 in costs within the first year with $184,891 annually, while veterans who still retain some motor function at all levels average $347,484 in costs within the first year and $42,206 annually^[48]^.

### Prescription medication coverage for those with TSCI

Considering the high cost of many prescription medications, 88% of veterans with TSCI obtain prescription medication coverage through the VHA. The remaining 12% utilize either a combination Medicare Part D & VHA (9.5%) or Part D alone (2.8%). This trend is likely to continue as most veterans with TSCI are exempt from medication co-payments through the VHA. Patients sustaining a TSCI or secondary comorbidity (i.e., pressure ulcer, UTI, diabetes) were found to rely less on Medicare Part D and more on the VHA for their prescription medication needs^[49]^.

## UNEMPLOYMENT AND BANKRUPTCY FOLLOWING TRAUMATIC SPINAL CORD INJURY

It is unsurprising that TSCIs of all severities are one of the most debilitating injuries a person can experience, often causing significant undue financial strain^[50]^. Despite many TSCI patients having a desire and capability to work, data show that only 35% of those having sustained TSCI eventually return to active employment^[51–53]^. Five years post-injury, 25% of these patients were found to file for bankruptcy^[54]^. TSCIs decrease the quality of life in patients due to their consequent inability to work and increased healthcare costs^[55]^. Following a TSCI, mobility/physical impairments and incontinence issues may limit the type of work available to TSCI patients^[56,57]^. Following a TSCI, skilled labor jobs may no longer be an option and many patients unable to return to their old jobs are forced to find new avenues of employment^[58,59]^.Realizing this difficulty, the Rehabilitation Act of 1973 was amended in 1992 to include supported employment (SE), which promotes disabled persons to return to the workforce. SE encourages those with significant disability to find jobs with competitive pay and have supportive services provided to those that in need^[60]^.

### Effectiveness of the supported employment initiative in veterans with TSCI

In 2012, a randomized multisite study investigated the effectiveness of a SE rehabilitation program in aiding military veterans with TSCI find post-injury employment. The initial results showed that veteran participants were 11.4 × more likely to find employment as compared to veterans without any form of rehabilitation program^[60]^. Two years later, a follow-up study was performed by the same investigators assessing the long-term performance of the previously studied SE rehabilitation program. The results showed that veterans were 30.8% more likely to achieve employment; however, veterans were significantly more likely to achieve employment within the first 12 months after their TSCI compared to those who waited longer than a year^[61]^.

In the same year as the 2-year follow-up study, a cost-effectiveness analysis was performed on the SE rehabilitation program for veterans with TSCI. Each participant received approximately 35 h of rehabilitation services costing $1,821 on average. The costs associated with the program were then compared to the quality of life improvement self-reported by each of the participants. The results showed that participants in the SE rehabilitation program had marginally reduced societal costs compared to the control group. But these results, coupled with an insignificant difference in quality of life improvement, led to the determination that the SE rehabilitation program was not cost-effective as compared to standard care.^[62]^

### Bankruptcy prevalence in those having sustained a TSCI

In the United States, the leading cause of bankruptcy is the inability to pay medical bills^[63]^. A study comparing the risk of bankruptcy before and after TSCI found that patients sustaining a TSCI have a 3.5% chance of bankruptcy in the first five years post-injury. Interestingly, those with private insurance were twice as likely to file for bankruptcy as compared to those with Medicaid. The authors attributed this finding to private insurance patients accruing additional debts pre-injury that they can no longer be paid back (i.e., car, mortgage, etc.)^[64]^. Race and income were also found play an important role for those returning to work post-injury. For caucasian patients it took a median of 566 days to return to work. However, their non-Caucasian counterparts took 1382 days to return to work, almost 2.5 times slower. Considering income, higher income patients in the upper 75th percentile returned to work in 557 days. In contrast, TSCI patients in the lower 25th percentile of income returned to work over 200 days later than their higher income counterparts. Phillips *et al*.^[58]^ attributed this delay to lower paying jobs often requiring skilled physical labor, causing an obvious barrier to TSCI patients.

Following a TSCI, patients report unemployment and financial difficulties as primary factors contributing to unhappiness, to a greater extent than the extent of their disability. Employment gives these patients a sense of both purpose and financial independence^[57]^. Patients with greater levels of social support, community integration and higher levels of education were more likely to gain steady employment^[65,66]^. TSCI may leave patients emotionally drained and separated from their social lives^[67]^. Prolonged unhappiness can exacerbate a variety of mental illnesses, with studies showing that 18%-37% of TSCI patients presented with signs of major depressive disorder (MDD)^[56]^. Financial stressors such as job loss, financial crisis, and inability to pay bills are found in 31.2% of those with MDD^[68,69]^. Patients following a TSCI may additionally have altered decision making capabilities due to the increased incidence of MDD^[70]^.

## CONCLUSION

TSCI is a lifelong costly injury for both hospital systems and the patient. Fast access to decompression surgery and early rehabilitation has been shown to improve injury outcomes. Although access to rehabilitation can be difficult through certain forms of insurance, it is critical in TSCI care. Lack of rehabilitation services has been associated with greater levels of comorbid secondary health conditions. Condition’s such as UTI, sepsis, and pressure ulcers account for higher health care cost utilization by TSCI patients. Reduction of secondary heath conditions is one of the few areas that can be modified in TSCI patients. Implementing rehabilitation and education about secondary health conditions for all TSCI patients would save both hospitals and patients money. These savings would allow for allocation of healthcare resources to other areas.

## Figures and Tables

**Table 1.** Percentage of patients with ASIA grade at ER discharge and resultant one year ASIA improvements

ASIA Grade	Injury type	Definition Of ASIA Grade	TSCI patients with ASIA Grade at time of Discharge*	ASIA Grade one year improvement rates (≥ 1 Grade level)**
Grade A	Complete	Complete sensorimotor loss	36.4%	25.1%
Grade B	Incomplete	Complete motor loss with incomplete sensory loss	13.8%	71.1%
Grade C	Incomplete	Motor function is preserved, but more than 50% of key muscles below the neurological level have a muscle grade < 3	11.9%	78.8%
Grade D	Incomplete	Motor function is preserved but the at least 50% of key muscles below the neurological level have a muscle grade ≥ 3	37.6%	14.1%
Grade E	Normal	Motor and sensory functions are normal	0.3%	N/A

ASIA: American spinal injury association

*: within each of the ASIA grade rows, there is the percentage of total TSCI patients at the time of hospital discharge with that specific ASIA grade injury out of all TSCI patients

ER: emergency room; TSCI: traumatic spinal cord injury

**: percentage of patients who have improved ≥ 1 ASIA grades from their original ASIA grade assignment (column 1) at one year post-discharge. Grade B and C injuries have the highest chance of improvements at 71.1% and 78.8%, respectively^[26,27]^
